# FluxFix: automatic isotopologue normalization for metabolic tracer analysis

**DOI:** 10.1186/s12859-016-1360-7

**Published:** 2016-11-25

**Authors:** Sophie Trefely, Peter Ashwell, Nathaniel W. Snyder

**Affiliations:** 1 AJ Drexel Autism Institute, Drexel University, Philadelphia, PA 19104 USA; 2 Department of Cancer Biology, Abramson Family Cancer Research Institute, University of Pennsylvania, Philadelphia, PA 19104 USA

**Keywords:** Metabolite, Correction, Tracer, Isotopologue, Enrichment, Flux, Normalization

## Abstract

**Background:**

Isotopic tracer analysis by mass spectrometry is a core technique for the study of metabolism. Isotopically labeled atoms from substrates, such as [^13^C]-labeled glucose, can be traced by their incorporation over time into specific metabolic products. Mass spectrometry is often used for the detection and differentiation of the isotopologues of each metabolite of interest. For meaningful interpretation, mass spectrometry data from metabolic tracer experiments must be corrected to account for the naturally occurring isotopologue distribution. The calculations required for this correction are time consuming and error prone and existing programs are often platform specific, non-intuitive, commercially licensed and/or limited in accuracy by using theoretical isotopologue distributions, which are prone to artifacts from noise or unresolved interfering signals.

**Results:**

Here we present FluxFix (http://fluxfix.science), an application freely available on the internet that quickly and reliably transforms signal intensity values into percent mole enrichment for each isotopologue measured. ‘Unlabeled’ data, representing the measured natural isotopologue distribution for a chosen analyte, is entered by the user. This data is used to generate a correction matrix according to a well-established algorithm. The correction matrix is applied to labeled data, also entered by the user, thus generating the corrected output data. FluxFix is compatible with direct copy and paste from spreadsheet applications including Excel (Microsoft) and Google sheets and automatically adjusts to account for input data dimensions. The program is simple, easy to use, agnostic to the mass spectrometry platform, generalizable to known or unknown metabolites, and can take input data from either a theoretical natural isotopologue distribution or an experimentally measured one.

**Conclusions:**

Our freely available web-based calculator, FluxFix (http://fluxfix.science), quickly and reliably corrects metabolic tracer data for natural isotopologue abundance enabling faster, more robust and easily accessible data analysis.

## Background

**Fig. 1:**
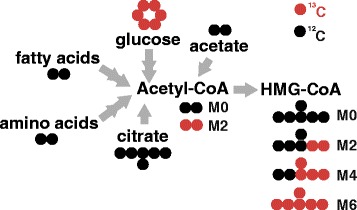
Incorporation of ^13^C-labeled substrate can be measured by mass changes in product metabolites. U-[^13^C_6_]-glucose incorporation into acetyl-CoA and subsequently into HMG-CoA is shown here as an example. Carbons derived from glucose can be incorporated into acetyl-CoA, and subsequently into HMG-CoA in units of 2. Thus, 2, 4 or 6 labeled carbons can be added to a HMG-CoA molecule, producing the M2, M4 or M6 isotopologues, respectively

Isotopic tracer analysis is a technique indispensable to the study of metabolic flux. A variety of stable isotopes are used for metabolic tracing depending on the purpose of the study. Stable isotopes are non-radioactive atoms with additional neutrons, and include ^13^C, ^15^N, ^18^O, and ^2^H. These ‘heavy’ isotopes possess chemical properties nearly identical to their lighter counterparts but differ in mass. The fate of isotope labeled atoms from substrates can be traced through their incorporation over time into specific metabolic products. The detection and differentiation of the isotopologues of each metabolite of interest is accomplished through mass spectrometry. Atoms from a labeled substrate can be incorporated singly or multiple times depending on the substrate and product being measured and the time frame considered, resulting in a distribution of isotopologues (molecules that differ only by their number of isotopic substitutions). The incorporation of [^13^C_6_]-glucose into acetyl-CoA and HMG-CoA is shown as an example (Fig. 1). The relative abundance of different combinations of ^13^C and ^12^C atoms (isotopologues) reflects the incorporation of the labeled substrate in competition with the other potential substrates.

Stable isotopes occur naturally on earth at varying rates. ^13^C carbon is an abundant naturally occurring isotope in biological systems. It is found on average at a rate of ~1.1% on the earth’s surface and in biological systems, although the carbon pool varies depending on its origin [1, 2]. The probability that an isotope of any atom will be incorporated into a molecule is determined by a number of factors including the elemental composition and the number of atoms present in the molecule. Incorporation of naturally occurring isotopes can make a significant contribution to molecular weight.

Relative quantitation of the different isotopologues of a metabolite must be adjusted for the natural background abundance of each isotopologue in order to make an accurate determination of artificial label incorporation. The normalization algorithm uses data from unlabeled samples, or from predicted isotopologue distribution using theoretical values from which the natural background isotopologue distribution can be estimated. This background distribution is then used to perform a transformation according to a well-established algorithm [3]. The output values indicate the enrichment of isotopologues derived from the artificially labeled substrate.

In practice this transformation is often performed as a series of calculations using software such as Excel (Microsoft), or via platform specific software. This method is prone to error due to the many steps involved and formulas requiring constant adjustment as data dimensions change for different metabolites. Programs capable of performing this calculation have been developed previously [3, 4] but they are implemented on software platforms that often suffer from compatibility, dependency and usability problems.

As HRMS technology improves to allow the acquisition of more metabolic tracer data, a bottleneck in experimental workflow is accentuated at the point of data analysis. In order to address this bottleneck and help streamline data analysis for metabolic tracer studies, we have developed FluxFix, an application freely available on the internet at http://fluxfix.science. FluxFix automatically performs the calculation from raw signal intensity values and converts them to percent molar enrichment values in one step. This program automatically adjusts to dataset dimension. FluxFix can be accessed at any time from any computer, overcoming the limitations of existing programs that are often platform specific, non-intuitive, commercially licensed and/or limited to using theoretical isotopologue distributions that can be prone to artifacts. Thus, it is robust, reduces error, is intuitive to the underlying data structure, more directly helps in interpretation, and saves time.

## Implementation

The application consists of a backend server running Ubuntu and an API written in Python 3.4.2 using numpy (https://github.com/numpy/numpy). The frontend was written in HTML, CSS, and makes use of Javascript. Altogether the program performs three functions as follows:

1. ‘Unlabeled’ data copy and pasted directly from spreadsheet applications including Excel (Microsoft) and Google sheets is read in as tab-separated values (TSV) and used to generate a correction matrix (M_Cor_). The website also includes an option to upload data in .CSV file format. Data must be formatted such that each row is a different sample and each column a different isotopologue. If more than one row of data is entered, the ‘unlabeled’ data is averaged over each column before generating M_Cor_.
2. Labeled sample data is read in as a data matrix (D_raw_) of several rows of TSV copy and pasted directly from a spreadsheet application or uploaded as a .CSV file. Data must be in the same format as the unlabeled data (i.e. each row is a different sample and each column a different isotopologue) and have the same column dimension. The corrected data (D_Cor_) is generated by convolving the correction matrix by the labeled data matrix as below:
  $$ {\mathrm{D}}_{\mathrm{Cor}}={\left({\mathrm{M}}_{\mathrm{Cor}}\right)}^{-1}.{\mathrm{D}}_{\mathrm{Raw}} $$
3. The percent molar enrichment for each isotopologue (column) is calculated for each individual sample (row). The output data is presented as a matrix of percent molar enrichment values in the same format as the input data matrix (D_raw_). The output appears in the results box as TSV and can be directly copy and pasted into a spreadsheet. The output can also be downloaded as a .CSV file.

The web interface has two boxes for data entry (unlabeled and labeled data) and another box for presenting computed results. The ‘Compute Percentages’ button runs the python program and generates the output data in the results box. The calculator can be instantly reset for new data entry by refreshing the page.

### User experience optimization

FluxFix was tested by release to a selected group of 20 users. These test users represented a range of levels of experience with isotopologue analysis and used a variety of differently structured datasets. After consultation with these users and acquiring feedback, we included several features that significantly improved user experience. These adaptations included:

- The dimensions of the data (x, y) are shown to the user upon input. For example “Data is ‘x’ columns by ‘y’ rows”. This helps the user to identify errors in data selection.
- Common errors in data input include the inadvertent entry of row/column headers and malformed data matrices (data can be malformed by the absence of a cell or the presence of extra cells as trailing tabs). These errors are caught by the client-side code and reported to the user as a pop-up prompt before sending. The pop-up prompt specifically describes the problem - either the presence of non-numeric values (row/column headers) or matrix malformation–it also describes the exact row and column coordinates of the error that triggered the report making it easy for the user to identify and rectify.
- If there is an error in processing the data on server-side, it is reported to the user by a pop-up prompt, which encourages them to contact us in the event of a persistent problem.

With ongoing user input and reporting, the usability of the application can be further improved. For programmers who might wish to implement the FluxFix calculation into an automated data analysis pipeline, a link is included on the webpage. This links to the project GitHub repository and instructions on how to implement the backend code in Python3 accompanied by example code.

## Results and discussion

Here we use example liquid chromatography mass spectrometry (LC-MS) data sets to demonstrate the application of FluxFix. We also outline recommendations for its application, and show the advantage of FluxFix through direct comparison to previously published isotope correction software.

### Example data set analysis

**Fig. 2:**
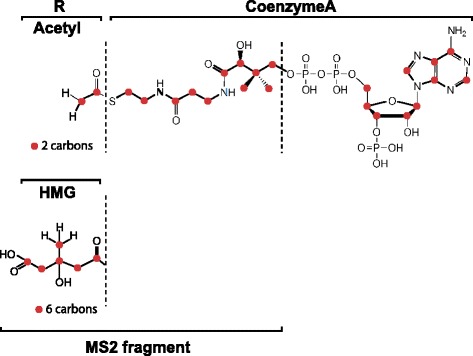
Molecular structure of acetyl-CoA and HMG-CoA. Carbon from glucose can be incorporated into the R-groups. The MS2 fragment measured experimentally incorporates the R-groups, as well as 11 other carbon molecules. Carbon atoms are highlighted as *red circles*

Here we demonstrate the application of FluxFix in the analysis of two different example datasets. The first example data set was generated as follows; HeLa cells were incubated in DMEM containing either 25 mM [^13^C_6_]-glucose or unlabeled glucose (for unlabeled control samples) for 4 h. MS data were acquired on a Thermo Q Exactive instrument in positive ESI mode as described elsewhere [5]. Quantitation was based on the relative abundance of MS2 fragments (Fig. 2). Processing of raw data and peak integration was performed using Xcalibur and TraceFinder (Thermo).

**Fig. 3:**
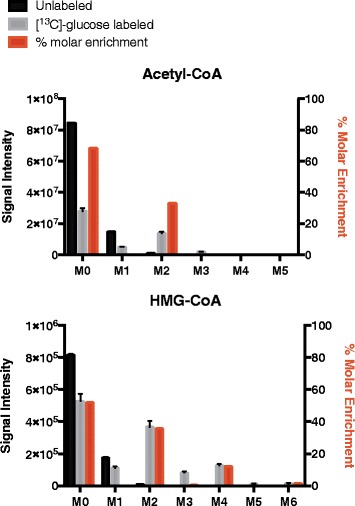
Data correction for acetyl-CoA and HMG-CoA using FluxFix. Input data as signal intensity (left y-axis) are in *black* and *grey* and output percent molar enrichment data (right y-axis) are in *red*. Molar enrichment from [^13^C]-glucose occurs in the M2 for acetyl-CoA and M2, M4 and M6 isotopologues for HMG-CoA. This incorporation of glucose is consistent with the known metabolic pathways by which glucose carbon is incorporated in pairs and to a maximum of two atoms for acetyl-CoA and six atoms for HMG-CoA. Data is from three replicate samples, error bars are standard deviation

The raw data and FluxFix output correction for the first example dataset are displayed in Table 1. This table includes a comparison of corrections derived from experimental unlabeled data and from theoretical unlabeled values. Theoretical values were generated for the MS2 fragments of acetyl-CoA and HMG-CoA (see Fig. 2) using the simulation function in XCalibur (Thermo). Figure 3 illustrates the correction using experimental data. It displays significant enrichment of the isotopologues (M0, M2, M4, M6) that can be derived from glucose, whilst the odd numbered isotopologues are not present. The metabolic pathways by which glucose is incorporated into acetyl-CoA and HMG-CoA require that it be added in two carbon units. Thus the exclusion of odd numbered isotopologues in the molar enrichment confirms the transformation was successful. The correction using simulated data results in more significant allocation of % molar enrichment to the odd numbered isotopologues, especially M1 (see Table 1), indicating that simulated unlabeled data may introduce more error.

**Table 1:** FluxFix correction for acetyl-CoA and HMG-CoA from [^13^C]-glucose treated cells. Output was generated with both simulated and experimental unlabeled data

Acetyl-CoA:	M0	M1	M2	M3	M4	M5	
Input: Signal Intensity Values							
unlabeled_1	8.45E + 07	1.48E + 07	7.38E + 05	2.35E + 04	0.00E + 00	0.00E + 00	
unlabeled_2	8.47E + 07	1.45E + 07	8.45E + 05	2.16E + 04	0.00E + 00	0.00E + 00	
unlabeled_3	8.41E + 07	1.49E + 07	9.58E + 05	3.09E + 04	0.00E + 00	0.00E + 00	
13C-Glc labeled_1	2.62E + 07	4.53E + 06	1.28E + 07	1.70E + 06	0.00E + 00	0.00E + 00	
13C-Glc labeled_2	2.73E + 07	4.88E + 06	1.38E + 07	1.81E + 06	0.00E + 00	0.00E + 00	
13C-Glc labeled_3	3.00E + 07	5.34E + 06	1.47E + 07	1.85E + 06	0.00E + 00	0.00E + 00	
unlabeled_simulation	809264.4	113786.2	36571.6	333.7	42.3	2.7	
Output: % molar enrichment (normalised to unlabeled data)							
labeled_1	68.63	−0.11	32.86	−1.30	−0.10	0.02	
13C-Glc labeled_2	67.68	0.29	33.48	−1.38	−0.10	0.02	
13C-Glc labeled_3	68.50	0.24	32.84	−1.53	−0.06	0.02	
Output: % molar enrichment (normalised to simulated data)							
13C-Glc labeled_1	68.73	2.22	30.16	0.09	−1.38	0.18	
13C-Glc labeled_2	67.78	2.59	30.84	0.01	−1.40	0.18	
13C-Glc labeled_3	68.60	2.57	30.15	−0.15	−1.35	0.18	
HMG-CoA:	M0	M1	M2	M3	M4	M5	M6
Input: Signal Intensity Values							
unlabeled_1	8.20E + 05	1.73E + 05	6.91E + 03	0.00E + 00	0.00E + 00	0.00E + 00	0.00E + 00
unlabeled_2	8.21E + 05	1.70E + 05	8.95E + 03	0.00E + 00	0.00E + 00	0.00E + 00	0.00E + 00
unlabeled_3	8.09E + 05	1.80E + 05	1.12E + 04	3.18E + 02	2.17E + 02	0.00E + 00	0.00E + 00
13C-Glc labeled_1	5.05E + 05	1.04E + 05	3.44E + 05	7.40E + 04	1.24E + 05	1.17E + 04	1.63E + 04
13C-Glc labeled_2	4.97E + 05	1.07E + 05	3.48E + 05	7.79E + 04	1.21E + 05	5.54E + 03	8.94E + 03
13C-Glc labeled_3	5.81E + 05	1.21E + 05	4.11E + 05	9.17E + 04	1.37E + 05	1.37E + 04	1.69E + 04
unlabeled_simulation	769011.9	141396.2	12234.4	661.6	25	0.7	0
Output: % molar enrichment (normalised to unlabeled data)							
13C-Glc labeled_1	52.29	−0.39	35.12	0.16	12.41	−1.44	1.86
13C-Glc labeled_2	52.12	0.09	35.90	0.50	12.18	−2.03	1.23
13C-Glc labeled_3	51.70	−0.27	36.06	0.46	11.69	−1.29	1.65
Output: % molar enrichment (normalised to simulated data)							
13C-Glc labeled_1	51.29	1.13	33.91	1.22	11.83	−1.04	1.66
13C-Glc labeled_2	51.12	1.61	34.69	1.57	11.60	−1.62	1.03
13C-Glc labeled_3	50.71	1.24	34.84	1.53	11.12	−0.90	1.46

The potential for isotope tracer analysis in metabolite discovery has attracted attention elsewhere [6]. Table 2 presents an example dataset that highlights the potential uses of FluxFix in metabolite discovery and characterization using mass isotopologue analysis. We make use of data from a previously published experiment of isotopologue analysis of an unknown product of propionate metabolism. This data was generated in human hepatocellular carcinoma HepG2 cells incubated in [^2^H_2_]-propionate or unlabeled propionate and was analyzed by MS/MS using an API-4000 triple quadrupole mass spectrometer, as described elsewhere [7]. Since, at the time of the experiment, the chemical formula of the putative metabolite was unknown, no generation of simulated spectra was possible. Therefore, an isotopic correction matrix was generated by treating a control group of cells with unlabeled sodium propionate. In Table 2, this data was used as input into FluxFix to calculate the percent molar enrichment of several isotopologues of the unknown compound.

**Table 2:** Isotopologue analysis of an unknown product of propionate metabolism. FluxFix generated percent molar enrichment output values from raw MS/MS data from cells treated with [^2^H_2_]-labeled or unlabeled propionate

SRM Transistion	864- > 357	865- > 358	866- > 359	867- > 360	868- > 361	869- > 362	870- > 363
Label	864_M0	864_M1	864_M2	864_M3	864_M4	864_M5	864_M6
Input: signal intensity values							
Prop_unlabeled_1	5.93E + 06	1.35E + 06	1.88E + 06	3.93E + 05	1.08E + 05	1.67E + 04	0.00E + 00
Prop_unlabeled_2	7.14E + 06	1.63E + 06	2.33E + 06	4.53E + 05	1.63E + 05	2.35E + 04	2.79E + 03
Prop_unlabeled_3	5.85E + 06	1.48E + 06	2.21E + 06	4.56E + 05	1.32E + 05	2.08E + 04	2.97E + 03
2H2-Prop_labeled_1	9.53E + 05	9.56E + 05	1.32E + 06	5.26E + 05	4.00E + 05	1.07E + 05	9.16E + 04
2H2-Prop_labeled_2	7.04E + 05	5.95E + 05	8.92E + 05	4.45E + 05	3.49E + 05	7.31E + 04	3.22E + 04
2H2-Prop_labeled_3	8.24E + 05	7.53E + 05	1.15E + 06	5.67E + 05	4.18E + 05	8.31E + 04	2.57E + 04
Output: % molar enrichment							
2H2-Prop_labeled_1	36.13	27.72	31.24	0.68	1.72	0.56	1.94
2H2-Prop_labeled_2	38.14	23.24	29.90	6.55	4.81	−2.07	−0.57
2H2-Prop_labeled_3	36.17	24.53	32.42	6.43	3.37	−2.20	−0.72

### Recommendations for use

The FluxFix calculator is flexible and can process input data derived from any type of isotope labeling strategy that can be analyzed by mass spectrometry and potentially from NMR spectra as well. We have tested FluxFix with a range of different datasets including glycolytic intermediates, acyl-CoA thioesters, lipids and novel metabolites. Furthermore, this program is not limited to ^13^C-labeled metabolites. Although we did not directly test this, FluxFix is compatible for use in conjunction with inductively coupled plasma-MS to measure incorporation of stable isotopes of elements as diverse as lead, calcium, iron, chromium, magnesium and zinc. FluxFix may also be used to analyze reverse labeling, or pulse-chase experiments, since the input data is label-neutral.

The principle recommendation we make is that experimentally derived data from unlabeled samples be used in preference to simulated background distribution data wherever possible. Relative isotopologue detection ([M + 1]/M) frequently diverges from theoretical values and this divergence is affected by numerous factors including instrument resolution [8, 9]. Simulated data is limited by its inability to account for matrix effects on resolution or to accurately represent background isotopic distributions unique to different biological systems.

In order to model isotopologue signal intensity values, one must model the resolution of the signal for every isotopologue included in the calculation. Theoretical isotopologue distribution is limited because there is no precise way to model matrix effects on resolution. Resolution is determined by a number of important factors. Firstly, the resolution of the instrument. Triple quadrupole and linear ion-trap instruments are often operated at unit resolution, but many have the ability to increase or decrease resolution. High-resolution mass analyzers operate with different constraints based on the underlying physics of ion detection and separation. Secondly, the resolution of an ion, in some mass analyzers is inversely dependent on the m/z of that ion. This dependency is not equivalent across platforms. For example, the decay in resolution with increasing m/z is not equivalent on an Orbitrap versus an Ion cyclotron resonance or time-of-flight instrument [10, 11]. Thirdly, resolution is dependent upon the sample matrix. Analytes are embedded in a matrix of ions, which varies according to the sample source and preparation. The proximity of neighboring ions (close in m/z) during acquisition of an analyte will directly influence the resolution of that analyte. These unique matrix effects cannot be consistently accounted for by theoretical predictions.

Different biological systems acquire unique isotopic signatures. Carbon fixation by C3 and C4 plants preferentially incorporate ^13^C at different rates [1]. Isotopes are propagated through the food chain such that species accumulate unique isotope signatures. This principle has been exploited in niche ecology, where variations in isotope profiles between organisms can be used to define food webs, diet, animal migration and nutrient flow [2, 12, 13].

Isotope tracer studies can be performed on samples from varied sources with unique background isotopologue distribution. Therefore, experimentally derived isotopologue distribution data, from unlabeled samples extracted in the same way as labeled samples, produce a more accurate representation of the ‘background’ isotopologue distribution than theoretical isotopologue distribution values. In light of this and the inability of simulations to account for matrix effects on resolution, we recommend that users of FluxFix use unlabeled sample data generated at least in triplicate from the matching matrix with the most experimentally relevant control conditions for normalization.

### Advantages of FluxFix over existing software

There are a number of available software platforms capable of performing isotopologue normalization. These include ICT [14] Pynac [15], (MS/)MS-X-Corr [16, 17], iMS2Flux [18], 13CFLUX2 [19], OpenFLUX [20], FiatFlux [21] and IsoCor [4]. With the exception of IsoCor, these are command line tools, which require an understanding of various programming languages (including Python, MatLab and Perl) and data structures to be used effectively. Many are designed to perform analysis on large ‘omics’ level data sets but are restricted to a single data acquisition platform or capable of detection of a single type of label incorporation eg ^13^C. A direct comparison of the features of several of these platforms can be found elsewhere [18].

FluxFix is unique as a web-based isotopologue normalization calculator. It performs a quick one-step calculation and does not require programming skills to use. The function of FluxFix is most similar to that performed by IsoCor [4] – a popular existing software platform. The major limitations of IsoCor are its use of theoretical isotope distribution, user-side software dependency, and inflexible data input requirements, upon which FluxFix improves. As the function of FluxFix is most similar to that of IsoCor, a detailed comparison of the features of these tools has been performed below.

- IsoCor is only available as a desktop software application with Python(x, y) dependencies and is only compatible with windows and Linux operating systems. FluxFix is available as a web application compatible with any modern web browser, eliminating the need for any software installation, configuration or compatibility issues. All that is required is an internet connection.
- IsoCor uses theoretical calculations to determine natural background isotopologue profiles. We do not encourage users to use simulations for background normalization. There are a variety of platforms, both free and proprietary, to simulate isotopologue distribution. For example, ChemCalc [22] is easily accessible and freely available as a web tool specifically designed for this purpose. If one choses to use simulated values routinely, we suggest that the user save them and use them in FluxFix, they need not be regenerated with every analysis, as with IsoCor.
- IsoCor requires input with stringent data dimensions based on the theoretical length of the isotopologue series. This data, although theoretically possible, is in practice rarely achieved owing to a requirement for extremely high sensitivity in acquisition. As a result, the user must add a series of zeros to the end of their detectable data peaks in order to satisfy the input data dimension requirements. The inflexible input data requirements can also lead to misleading results as any isotopologues that might be invalid for acquisition reasons (e.g. the resolution was bad and contaminated with interfering peaks) cannot be omitted from the data set. FluxFix adapts to the input data dimensions chosen by the user.
- IsoCor takes input as an exported .txt file that the user must generate. This extra step is not required in FluxFix, which streamlines direct copy and paste from spreadsheet applications including Excel (Microsoft), saving time and processing effort.
- Additionally, IsoCor output data for batch analyses, is as a separate data .txt file. FluxFix presents the output in the results window in the same format as the input data matrix, facilitating direct copy and paste into a spreadsheet and a faster workflow.

IsoCor incorporates features that are not included in FluxFix. These are the residuum score, the derivatization feature and the isotope purity correction. We argue that these features are superfluous to an effective data workflow and could lead to data overcorrection.

- IsoCor relies on a user editable file that details the isotope percent enrichment for each of the atoms being analyzed. This data is used to simulate the isotopologue distribution. FluxFix does not perform these simulations as we encourage the use of real unlabeled sample data for normalization. However, as described above, simulated isotopologue distributions can be generated for any specific chemical structures using a variety of existing software options.
- IsoCor has a function that adds a derivatization group to the calculation for isotopologue distribution. This can lead to confusion because there are many different chemical structures that can be produced from a derivatized parent molecule. In isotopologue analyses, one must be specific about the chemical structure being analyzed. FluxFix relies on the user defining the isotopologue masses detected, making it clearer and more flexible.
- Negative values are theoretically impossible but often occur in small values owing to variability and error. IsoCor incorporates an algorithm that penalizes negative values upon normalization such that the error involved in this penalty is reflected, instead, in a residuum score. FluxFix does not perform a penalty or give residuum scores. We argue that this penalty can be misleading, as it masks the error evident in negative values making it less likely that the user is alerted to inconsistencies in their data.
- IsoCor has an option to correct for the purity of the isotope tracer used in an experiment. FluxFix does not perform this calculation, as it is not required. The purpose of FluxFix is to calculate the enrichment of label incorporation above naturally occurring isotopes. For most isotope labeling experiments a substrate is used with an approximate purity measure designated by the manufacturer (usually ~98%). The exact purity is not actually known, therefore correcting for this factor is not useful and could actually over-correct the data.

The simplicity and ease of use of FluxFix separates it from previous software for metabolic tracer correction. FluxFix is flexible both in its availability (online at any time) and in its input parameters – it can process data in the most easily accessible format (TSV from spreadsheet) and for any metabolite for which isotopologue data has been generated, there are no limitations on the dimensions of this data. It is not restricted to particular isotopes or by settings for a limited range of isotopes. Thus, FluxFix can be easily applied to an unlimited range of metabolites. Finally, the program has near seamless integration with spreadsheet applications including Microsoft excel and Google sheets, which helps the user organize data. In addition, the built-in checks for data compatibility and notation of the dimensions of the data as they are pasted in assist in error proofing.

## Conclusions

Our freely available web based calculator, FluxFix (http://fluxfix.science), quickly and reliably corrects metabolic tracer data for natural isotopologue abundance enabling faster, more robust data analysis. It is flexible, accurate, and can be used for any tracer, any metabolite, by any computer with an Internet connection. Thus it is a simple, convenient and flexible solution to the data bottleneck problem in metabolic tracer analysis.

## Abbreviations

HMG-CoA: 3-hydroxy-3-methyl-glutaryl-Coenzyme A
TSV: Tab-separated values
